# Characterisation and microleakage of a new hydrophilic fissure sealant - UltraSeal XT^®^ hydro™

**DOI:** 10.1590/1678-775720160010

**Published:** 2016

**Authors:** Zeynep A. GÜÇLÜ, Nazmiye DÖNMEZ, Andrew P. HURT, Nichola J. COLEMAN

**Affiliations:** 1 - Erciyes Üniversitesi, Diş Hekimliği Fakültesi Pedodonti, Kayseri, Turkey.; 2 - Bezmialem Vakıf Üniversitesi, Diş Hastalıkları ve Tedavisi, İstanbul, Turkey.; 3 - University of Greenwich, Faculty of Engineering and Science, Kent, United Kingdom.

**Keywords:** Hydrophilic, Fissure sealants, Er:YAG lasers, Microleakage, Microhardness

## Abstract

**Objectives:**

The aim of this study was to characterise the new hydrophilic fissure sealant, UltraSeal XT^®^ hydro™ (Ultradent Products, USA), and to investigate its *in vitro* resistance to microleakage after placement on conventionally acid etched and sequentially lased and acid etched molars.

**Material and Methods:**

The sealant was characterised by Fourier transform infra-red spectroscopy (FTIR), scanning electron microscopy (SEM), energy dispersive X-ray analysis (EDX), and Vickers indentation test. Occlusal surfaces of extracted human molars were either conventionally acid etched (n=10), or sequentially acid etched and laser irradiated (n=10). UltraSeal XT^®^ hydro™ was applied to both groups of teeth which were then subjected to 2,500 thermocycles between 5 and 55°C prior to microleakage assessment by fuchsin dye penetration.

**Results:**

UltraSeal XT^®^ hydro™ is an acrylate-based sealant that achieved a degree of conversion of 50.6±2.2% and a Vickers microhardness of 24.2±1.5 under standard light curing (1,000 mWcm^-2^ for 20 s). Fluoride ion release is negligible within a 14-day period. SEM and EDX analyses indicated that the sealant comprises irregular submicron and nano-sized silicon-, barium-, and aluminium-bearing filler phases embedded in a ductile matrix. Laser preconditioning was found to significantly reduce microleakage (Mann-Whitney U test, p<0.001). The lased teeth presented enhanced surface roughness on a 50 to 100 μm scale that caused the segregation and concentration of the filler particles at the enamel-sealant interface.

**Conclusion:**

Laser preconditioning significantly decreased microleakage and increased enamel surface roughness, which caused zoning of the filler particles at the enamel-sealant interface.

## INTRODUCTION

Approximately 90% of all dental caries arise in the occlusal pits and fissures, since these regions are anatomically defended against the remineralising flow of saliva and routine brushing^8^. Accordingly, resin-based or glass ionomer sealants are placed over the occlusal surfaces of premolars and molars to prevent cariogenic microorganisms and fermentable organic debris from accumulating in the pits and fissures^11^.

The majority of commercially available resin-based pit and fissure sealants are hydrophobic materials that bond to the enamel surface via micromechanically interlocking tags^13^. The presence of moisture and saliva-contamination during the placement of the sealant compromise the quality of adhesion at the sealant-enamel interface, which impacts the ongoing resistance to microleakage of microorganisms. Recently introduced hydrophilic sealants, which bond effectively to moist enamel surfaces, present a distinct advantage in paediatric dentistry where patient-compliance, isolation, and moisture-control can be particularly challenging^10^.

UltraSeal XT^®^ hydro™ is a new moisture-tolerant, self-adhesive, light-cured, acrylate-based, hydrophilic pit, and fissure sealant which has been developed by Ultradent Products, USA^4^. The sealant comprises a 53 wt% mixture of inorganic filler particles that confer radiopacity. This material is reported to “chase” moisture into the pits and fissures, thus eliminating moisture-related failure associated with hydrophobic sealants^4^.

Prior to the application of a resin-based fissure sealant, the enamel surface is etched with phosphoric acid gel to enhance surface roughness and wettability and to increase porosity and surface area available for bonding^1^. Recent research has indicated that the application of laser ablation as an adjunct to phosphoric acid etching may improve the adhesion, marginal adaptation, retention, and resistance to microleakage of resin-based sealants^10^. This finding is not universal, and the efficacy of laser conditioning appears to depend upon the rheological and physicochemical properties of different sealants^3,5,12^. It is suggested that less viscous, more “flowable” materials may afford better adaptation to the rougher surfaces presented by lased enamel^9^.

The objectives of this study were to characterise the new hydrophilic sealant, UltraSeal XT^®^ hydro™, and to investigate its resistance to microleakage *in vitro*. The chemical composition and degree of curing of UltraSeal XT^®^ hydro^™^ were investigated by Fourier transform infra-red spectroscopy (FTIR). Scanning electron microscopy (SEM) was used to examine the fracture surface and the elemental composition was determined by energy dispersive X-ray analysis (EDX). Microhardness was evaluated using the Vickers indentation test, and fluoride-release was monitored with an ion-selective electrode.

## MATERIAL AND METHODS

Ethical approval for this project was obtained on 1^st^ October 2014 by the Ethical Committee of Bezmiâlem Vakif University (reference number 71306642/050-01-04/282), which was performed according to the ethical standards laid down in the 1964 Declaration of Helsinki and its later amendments.

### Characterisation of UltraSeal XT® hydro™

**Figure 1:**
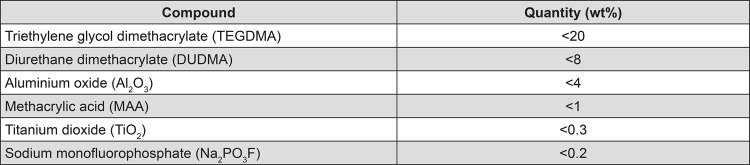
Composition of UltraSeal XT® hydro™

The composition of the UltraSeal XT^®^ hydro^TM^ sealant (Ultradent Products, South Jordan, Utah, USA) used in this study, as listed in the safety data sheet provided by the manufacturer^14^, is given in Figure 1. Cylindrical polypropylene moulds of 7 mm diameter and 2 mm depth were filled with UltraSeal XT^®^ hydro^™^ monomer solution that was light-cured for 20 s at 1000 mWcm^-2^ (using a BA Optima 10 curing light, BA International Ltd., Northampton, Northamptonshire, UK). The resulting cured UltraSeal XT^®^ hydro^™^ discs were then characterised by FTIR, SEM, and EDX and their microhardness and fluoride-release behaviour were measured.

### Scanning electron microscopy and energy dispersive X-ray analysis

Fracture surfaces of UltraSeal XT^®^ hydro^™^ for SEM and EDX analysis were produced by crushing the cured discs between steel plates under a low-impact force. Scanning electron microscopy was carried out on the fracture surfaces of uncoated samples attached to carbon tabs on a JEOL JSM-5410 LV electron microscope with an Oxford Instruments X-MaxN EDX detector in low vacuum mode. All back-scattered and secondary electron images were obtained with an accelerating voltage of 1.0 kV at a working distance of 8.4 mm.

### Fourier transform infra-red spectroscopy

The FTIR spectra of the unset UltraSeal XT^®^ hydro^™^ monomer solution and of the cured discs were obtained in triplicate using a Perkin Elmer Spectrum Two spectrometer with a Universal Diamond attenuated total reflectance attachment (Perkin Elmer, London, UK). Spectra were recorded with 16 accumulated scans between 4000 cm^-1^ and 450 cm^-1^ wavenumbers at a resolution of 4 cm^-1^.

The degree of conversion (DC) was estimated by comparing the ratios of the intensities of the FTIR peaks for the reactive polymerising C=C bond (at 1637 cm^-1^) and the invariant C=O bond (at 1717 cm^-1^) in the cured polymer and monomer using the following equation:


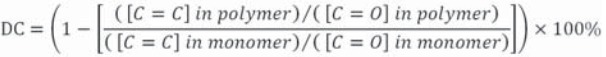


### Fluoride ion release

Three cured UltraSeal XT^®^ hydro^™^ discs were individually placed in three polypropylene centrifuge tubes containing 5 cm^3^ of deionized water and stored in the absence of light at 37°C. The release of free fluoride ions from the cured discs was monitored daily throughout a 14-day period using an ion-selective electrode (Cole-Parmer, London, UK) and Orion 4 Star meter (Thermo Fisher Scientific, East Grinstead, UK).

### Microhardness evaluation

The Vickers microindentation test was carried out on 3 UltraSeal XT^®^ hydro^™^ discs using a Buehler Micromet Hardness Tester (Buehler, Coventry, Warwickshire, UK) with a load of 200 g and contact time of 2.5 s. Three measurements were taken at random points on both sides of each sample disc. Indentation diagonals were measured in micrometres with the aid of a stereomicroscope and converted to Vickers hardness numbers (VHN) using conversion tables provided by Buehler, Coventry, Warwickshire, UK.

### Microleakage assessment

**Figure 2:**
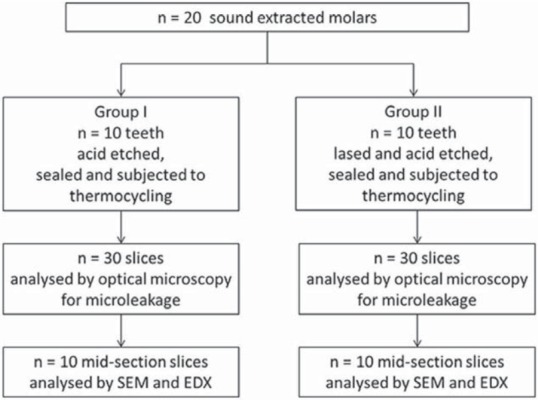
Flow diagram of experimental procedures

Twenty sound extracted human molar teeth were obtained from patients with orthodontic or periodontal problems who had tendered their informed consent. The teeth were manually debrided with scaling instruments, cleaned with pumice paste and stored in distilled water for up to four weeks. The teeth were then randomly divided into two groups. A flow diagram for the experimental procedures is given in Figure 2.

The occlusal surfaces of Group I teeth (n=10) were acid etched with 35% phosphoric acid gel (UltraSeal XT^®^ hydro^™^, Ultradent Products, South Jordan, Utah, USA) for 20 s, rinsed and lightly air dried, as suggested by the manufacturer. The UltraSeal XT^®^ hydro^™^ sealant was then applied by the same operator, according to the manufacturer’s instructions, and light cured for 20 s (using a BA Optima 10 curing light, BA International Ltd., Northampton, Northamptonshire, UK).

Group II teeth (n=10) were sequentially subjected to laser ablation and acid etching. Laser conditioning of the occlusal surfaces of Group II teeth was carried out using a 2940 nm Er:YAG laser system (LightWalker^®^, Fotona, Ljubljana, Slovenia). The Er:YAG laser energy was applied at a density of 19 mJ cm^-2^, power output of 1.2 W, and pulse energy of 120 mJ using a 600 μm diameter sapphire tip with a beam spot size of 0.63 mm^2^ at a working distance of 8 mm at an angle of 90° under water cooling at 50 cm^3^min^-1^. The lased teeth were rinsed with water, thoroughly air dried, acid etched (as outlined above), and then lightly air dried prior to sealing with UltraSeal XT^®^ hydro^™^.

**Figure 3:**
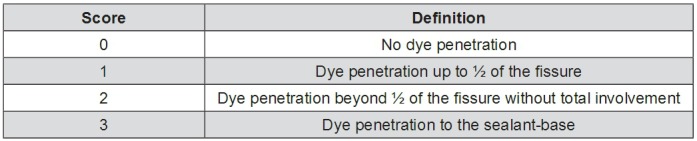
Microleakage scoring criteria

Immediately after sealing, the teeth were placed in distilled water at 37°C for 24 h and then thermocycled 2500 times between 5 and 55°C with a transfer time of 10 s and a dwell time of 30 s. The teeth were then coated with nail varnish, leaving a 2 mm window around the sealant, and the roots were embedded in an acrylic resin cylinder (Meliodent, Bayer Co., Leverkusen, Germany). Each sample was placed in 0.5% basic fuchsin dye solution for 24 h, rinsed under flowing tap water for 5 min to remove excess dye, and sectioned in the bucco-lingual direction using a water-cooled diamond saw to obtain three slices. Each of the tooth sample slices was then examined twice under a stereomicroscope (SMZ 800, Nikon, Tokyo, Japan) at 20x magnification by two investigators who were unaware of the pre-treatment of each sample. The microleakage scoring criteria are listed in Figure 3 ^1,9^.

The microleakage data were analysed using the statistical package SPSS 14.0.0 for Windows (SPSS, Chicago, Illinois, USA). Significant differences were evaluated using the Mann-Whitney U test (p=0.05). Inter-examiner variability was analysed with the kappa statistic, which was found to be 0.9, indicating high reproducibility.

### SEM and EDX analyses of sealed teeth

SEM and EDX analyses were carried out on the middle slices of the sectioned teeth using uncoated samples attached to carbon tabs on a JEOL JSM-5410 LV electron microscope with an Oxford Instruments X-MaxN EDX detector in low vacuum mode. All back-scattered electron images and EDX maps were obtained with an accelerating voltage of 20 kV at a working distance of 20 mm.

## RESULTS

### SEM and EDX analyses

**Figure 4:**
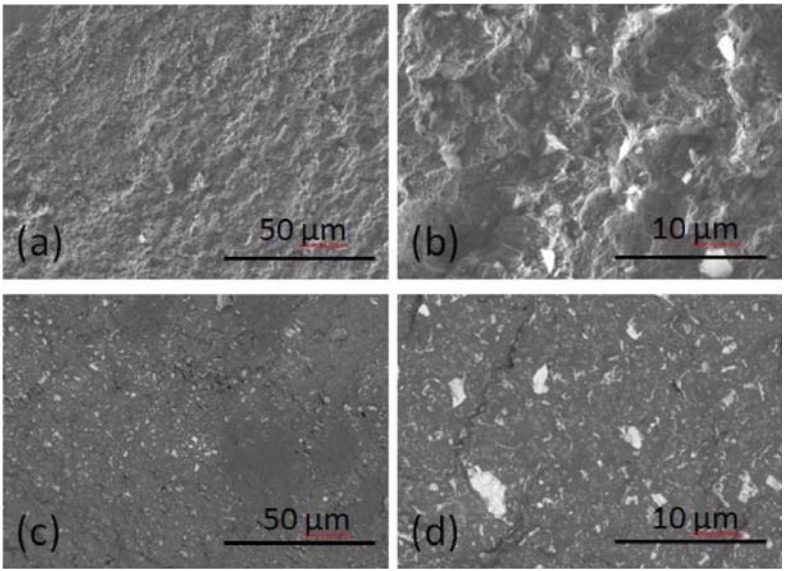
Fracture surfaces of UltraSeal XT® hydro™ as (a,b) secondary electron images and (c,d) back-scattered electron images

Secondary electron images showing the topography of the fracture surfaces of light cured UltraSeal XT^®^ hydro^™^ are presented in Figures 4a and 4b. Angular filler fragments of up to ~5 μm in diameter are seen to be embedded in the polymerised matrix. The textured irregular surfaces observed arise from ductile fracture with associated plastic deformation.

Back-scattered electron images of the UltraSeal XT^®^ hydro^™^ fracture surfaces (Figures 4c and d) provide compositional information, since elements with higher atomic number scatter electrons more effectively and appear as brighter regions. These images also show a distribution of highly angular and fibrous submicron and nano-sized filler particles throughout the polymerised matrix.

The EDX analysis of a 100 μm by 125 μm window of a polished section of the light cured sealant indicated that this material is principally composed of carbon, oxygen, barium, silicon, aluminium, calcium, and phosphorus (Table 1). Individual EDX spot analyses of the filler particles showed that these phases comprise a mixture of silicon-, aluminium-, and barium-bearing minerals. Sodium was also noted at approximately 0.1 wt%. The manufacturer’s safety data sheet for UltraSeal XT^®^ hydro^™^ (Figure 1 ^14^) lists sodium monofluorophosphate (Na_2_PO_3_F) at levels below 0.2 wt%, which were found to be below the EDX detection limit for fluorine.

**Table 1:** Elemental composition of UltraSeal XT® hydro™

Element	C	O	Ba	Si	Al	Ca	P
Mass* (wt%)	44	30	9.6	7.7	4.7	2.4	1.8

*Relative standard deviations are less than 2% in each case

### FTIR spectroscopy

**Figure 5:**
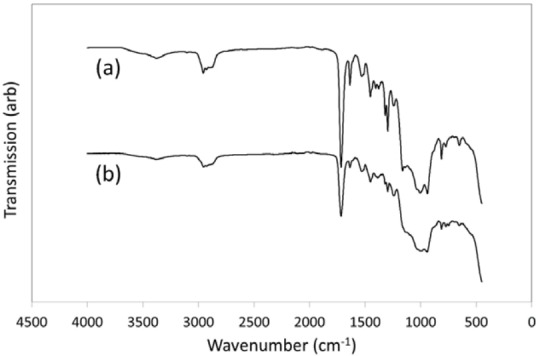
FTIR spectra of (a) unset UltraSeal XT® hydro™ and (b) light-cured UltraSeal XT® hydro™

**Figure 6:**
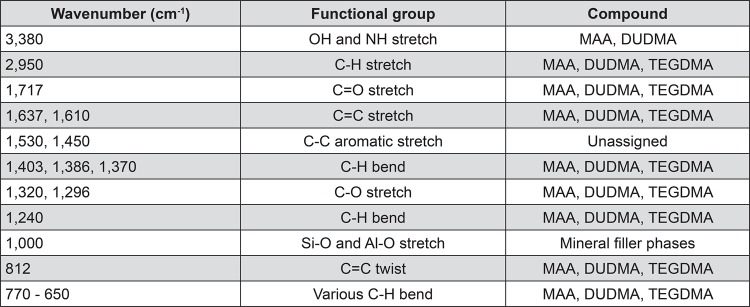
Functional group assignments for the FTIR spectra of un-cured and cured UltraSeal XT® hydro™

The FTIR spectra of the monomeric UltraSeal XT^®^ hydro^™^ solution and light cured polymerised discs are shown in Figure 5, and the corresponding functional group assignments are listed in Figure 6 ^15^. According to the manufacturer’s safety data sheet (Figure 1 ^14^), the organic component of UltraSeal XT^®^ hydro^™^ comprises a mixture of triethylene glycol dimethacrylate (TEGDMA), diurethane dimethacrylate (DUDMA), and methacrylic acid (MAA). Present in these monomers are hydrophilic carboxylic acid (-COOH), secondary amine (-NH) and carbonyl (-C=O) groups, hydrophobic alkane (-CH_3_) and alkene (-C=CH_2_) groups, and ether (-C-O-C-) groups, which are of amphiphilic character. Each of these functional groups appears in the FTIR spectra of both the unset UltraSeal XT^®^ hydro^™^ solution and light cured polymerised discs (as indicated in Figure 5). In addition to these groups, aromatic carbon-carbon stretching vibrations are also present in the FTIR spectra of both unset and light cured UltraSeal XT^®^ hydro™, which indicate the presence of undisclosed aromatic monomer and/or initiator compounds within the mixture.

The very broad compound signal centred at approximately 1000 cm^-1^ wavenumbers in the unset and light cured FTIR spectra of the sealant arises from various Si-O and Al-O stretching modes of the silicon- and aluminium-bearing mineral filler phases.

The degree of conversion of resin-based sealants is defined as the percentage of acrylate C=C groups from the various monomers that have undergone polymerisation^7^. The mechanical, physical, and chemical integrity and clinical performance of the cured sealant are strongly dependent upon this property. Analysis of the FTIR spectra of unset and light cured UltraSeal XT^®^ hydro^™^ indicated that this sealant achieved a degree of conversion of 50.6±2.2% under the selected experimental conditions.

### Fluoride ion release

Light cured UltraSeal XT^®^ hydro^™^ discs failed to release any detectible free fluoride ions into deionised water during a 14-day observation period, indicating that any fluoride ion release was at a concentration below 0.001 ppm. According to the manufacturer’s safety data sheet (Figure 1 ^14^), the level of sodium monofluorophosphate (Na_2_PO_3_F) in UltraSeal XT^®^ hydro^™^ is lower than 0.2 wt%. This corresponds with a maximum fluoride ion concentration within the sealant of 0.0264 wt%.

### Microhardness

Microhardness provides a measure of the resistance of a sealant to plastic deformation under applied compressive forces. The Vickers hardness number (VHN) of light cured UltraSeal XT^®^ hydro^™^ was found to be 24.2±1.5.

### Microleakage assessment

The distributions of microleakage scores for the acid etched (Group I) and sequentially lased and acid etched (Group II) teeth are listed in Table 2. Thirteen of the Group I teeth sections exhibited microleakage with maximum dye penetration to the sealant base; whereas, only one Group II tooth section demonstrated microleakage within the upper half of the fissure. These data indicate that the application of Er:YAG laser ablation under the selected experimental conditions prior to acid etching significantly improves the *in vitro* resistance of the sealed teeth to microleakage (p<0.001).

**Table 2:** Microleakage scores as functions of enamel conditioning

Microleakage score	0	1	2	3	*Significance
Group I (Acid)	17	3	5	5	a
Group II (Laser and acid)	29	1	0	0	b

*Different letters indicate significant differences (p<0.001)

### SEM and EDX analyses of sealed teeth

**Figure 7:**
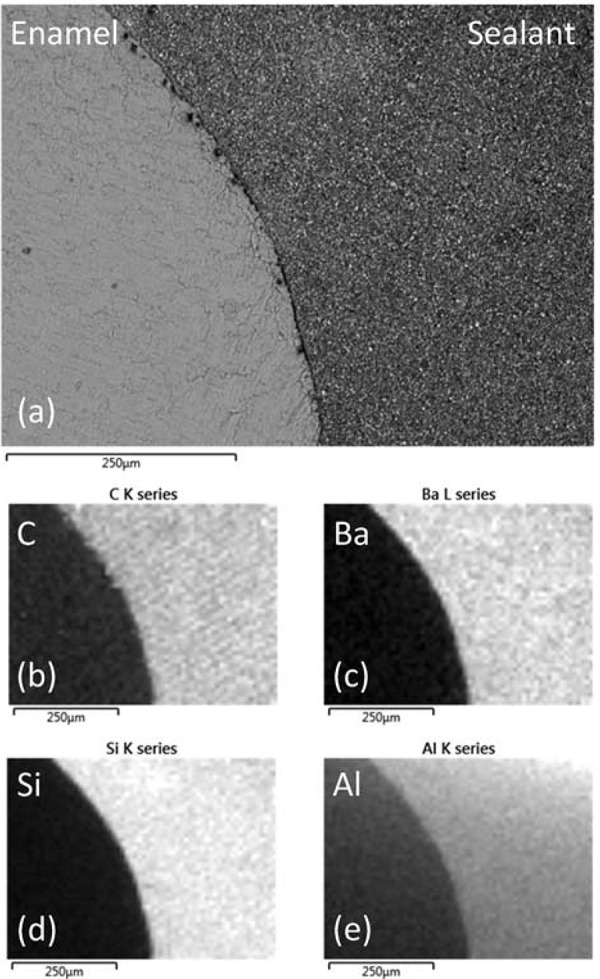
(a)- Back-scattered scanning electron microscopy image of UltraSeal XT® hydro™ in contact with acid-etched enamel and corresponding EDX maps of (b) carbon, (c) barium, (d) silicon, and (e) aluminium

**Figure 8:**
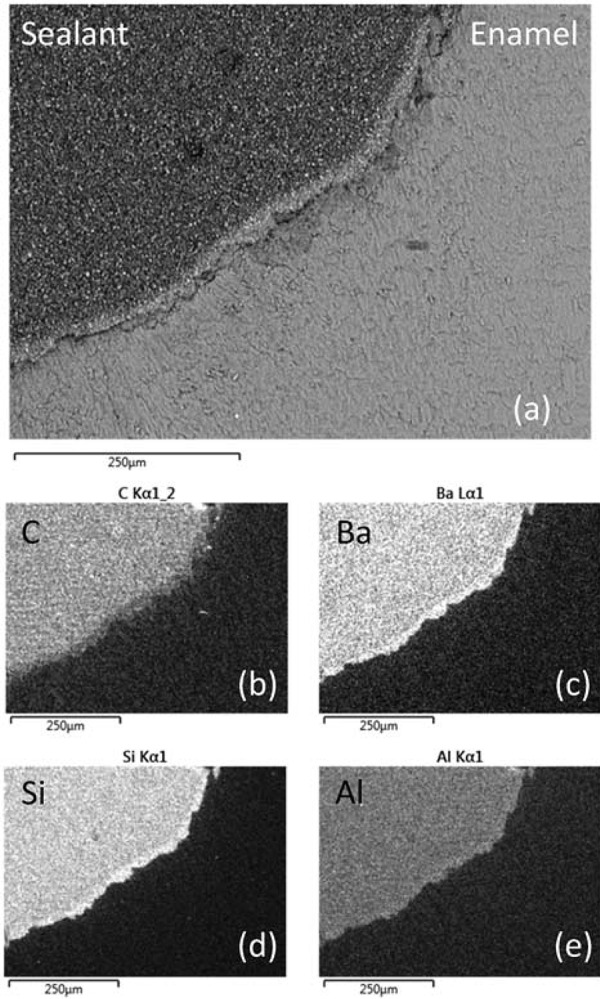
(a) Back-scattered scanning electron microscopy image of UltraSeal XT® hydro™ in contact with sequentially lased and acid etched enamel and corresponding EDX maps of (b) carbon, (c) barium, (d) silicon, and (e) aluminium

Back-scattered electron images of the enamel-sealant interfaces of acid etched (Group I) and sequentially lased and acid etched (Group II) teeth are shown in Figures 7a and 8a. Enhanced surface roughness on a 50 to 100 μm scale was observed for the enamel surfaces that had been preconditioned with the Er:YAG laser. This observation confirms those of other studies which report enhanced surface roughness of up to 155 μm for laser conditioned teeth^1,9^. The filler within the sealant was observed to remain homogeneously distributed when the material was placed in contact with the acid etched enamel surface; however, the enhanced surface roughness of the lased teeth caused the segregation and concentration of the filler particles at the enamel-sealant interface. Corresponding elemental carbon, barium, silicon, and aluminium EDX maps of the sealed Group I and II teeth are presented in Figures 7(b,c,d,e) and 8(b,c,d,e), respectively. These images highlight the zoning of the barium-, silicon-, and aluminium-bearing mineral phases at the enamel-sealant interface of the lased teeth.

## DISCUSSION

### Characterisation

Scanning electron microscopy with EDX analysis of fracture surfaces of UltraSeal XT^®^ hydro™ demonstrated that this sealant is a ductile material that is highly filled with uniformly distributed micrometre and nanometre sized silicon-, aluminium-, and barium-bearing mineral phases. This finding contradicts the information contained in the manufacturer’s safety data sheet that indicates that this sealant contains aluminium and titanium oxides and does not list constituent silicon- and barium-bearing mineral phases (Figure 1)^14^. It should be noted that, despite the manufacturer’s claim, no titanium phases were detected in the sealant by EDX analysis.

The curing efficiency of this sealant, ~51%, appears towards the lower end of the range (~50 to 76%) reported for contemporary commercially available resin-based fissure sealants cured under similar experimental conditions^6,11^. It is considered that the high level of inorganic mineral filler in UltraSeal XT^®^ hydro^™^ may impact the degree of cure by inhibiting light penetration and also by presenting a physical barrier to monomer cross-linking.

The microhardness of UltraSeal XT^®^ hydro^™^ (VHN ~24) was found to fall within the range reported for commercial resin-based sealants (VHN from ~9 to 75)^2,6,11^. In general, despite the reduced curing efficiency of highly filled resin-based sealants, microhardness tends to increase with increasing filler-content^11^. In this respect, submicron and nano-sized fillers, such as those present in this sealant, are regarded to be particularly effective in increasing microhardness and reducing plastic deformation under occlusal forces^11^.

Any fluoride species present in UltraSeal XT^®^ hydro^™^ were found to be below the detection limits of both the EDX instrument and the ion-selective electrode. In contrast, during a 30-day period under similar experimental conditions, other commercially available resin-based fissure sealants are reported to release up to 58 μg cm^-2^ of free fluoride ions^11^. However, these reported levels of fluoride release are unlikely to be significant regarding any beneficial impact on acid- and caries-resistance of local enamel, and, in this respect, the lack of detectible fluoride release from UltraSeal XT^®^ hydro™ is not regarded to be a comparative disadvantage.

### Microleakage

Data obtained under the selected experimental parameters in this study indicate that laser ablation prior to conventional acid etching significantly improves the *in vitro* resistance of the sealed teeth to microleakage (Table 2). The superior resistance to microleakage of the lased teeth is attributed to the enhanced roughness of the ablated enamel surfaces. This finding confirms that of Khogli, et al.^10^ (2013) who investigated the impact of laser ablation as an adjunct to acid etching to precondition molars prior to sealing with a filled hydrophilic sealant. Other studies, however, report that lasing prior to acid etching provides no statistically significant improvement in resistance to microleakage^3,5^. This lack of consensus on the effectiveness of Er:YAG laser ablation prior to acid etching is likely to arise from the range of laser settings employed by various researchers and the suitability of different sealants to the lased enamel surfaces^3,5,10^.

The principal mode of adhesion of resin-based fissure sealants to enamel is via the formation of micromechanically interlocking tags that penetrate the macro- and micro-porosities presented by the preconditioned enamel. Very little physicochemical interaction exists between the high energy surface of the hydroxyapatite in enamel and the organic constituents of the sealant^13^.

Enamel preconditioning by etching with phosphoric acid gel is currently the most common method used to prepare the occlusal tooth surface to receive the fissure sealant. This method of etching may not prove entirely effective on aprismatic enamel located at the fissure entrance and in the presence of remnant pellicle biofilm and organic debris lodged in deep narrow fissures^1,5,9^. Potential advantages of laser ablation prior to acid etching are reported to be: enhanced roughness and wettability of the lased enamel surface; more effective removal of pellicle and debris; superior conditioning of aprismatic hydroxyapatite; and enhanced acid-resistance of lased enamel^5,9^. Disadvantages of laser conditioning include the potential vitrification of the enamel surface at subablative laser energies and excessive roughness and microcracking under extreme ablation^5,12^. Laser conditioning of enamel is a relatively recent technique and further research is required to optimise the operating parameters.

It is conjectured that the more viscous fissure sealants may fail to flow readily and fully adapt to the rougher surfaces presented by lased enamel^10^. In addition, this study has demonstrated that inorganic filler particles within a sealant may partially separate out from the resin phase and congregate at the interface as it flows over the rough lased surface. It is not presently known whether this congregation of filler particles at the interface of lased enamel will have a detrimental impact on the long-term clinical performance of the sealant.

At present, all commercial resin-based fissure sealants are designed for acid etched enamel. It is suggested that, to take full advantage of the potential benefits of laser conditioning, a new generation of fissure sealants, which are specifically tailored to adhere and adapt to lased enamel, is required.

## CONCLUSIONS

The purpose of this study was to characterise the new resin-based hydrophilic fissure sealant, UltraSeal XT^®^ hydro™ (Ultradent Products, USA), and to investigate its *in vitro* resistance to microleakage after placement on conventionally acid etched and sequentially lased and acid etched molars.

Scanning electron microscopy and energy dispersive X-ray analysis of fracture and polished surfaces indicated that UltraSeal XT^®^ hydro™ comprises irregular submicron and nano-sized silicon-, barium-, and aluminium-bearing filler phases embedded in a ductile resin matrix. Under standard light curing, UltraSeal XT^®^ hydro™ achieved a degree of conversion (50.6±2.2%) and a Vickers microhardness (24.2±1.5) towards the lower end of the reported ranges for other commercial acrylate-based fissure sealants.

Laser preconditioning was found to significantly decrease microleakage and to increase enamel surface roughness, which caused zoning of the filler particles at the enamel-sealant interface.
